# Double Fire Tragedy of Kenya

**Published:** 2009-12-16

**Authors:** Lester Young, Rowena Orosco, Stephen Milner

**Affiliations:** Johns Hopkins Burn Center, Johns Hopkins University School of Medicine, Baltimore, Maryland

## Abstract

**Objective**: Within days of each other, 2 catastrophic fires occurred in Kenya. On January 28, 2009, a busy supermarket was destroyed in downtown Nairobi. Shortly thereafter on February 2, an overturned petrol tanker exploded near the village of Molo, 200 km from the capital. These 2 disasters, in an urban and a rural setting, respectively, illustrate the lack of disaster readiness on a local and national level. **Methods**: A call for assistance was responded to by the James Jordan Foundation, which sponsored a team from the United States to provide consultation and patient care. Subsequent to this team's experiences, a review of medical records at the Kenyatta National Hospital, interactions with government health officials, and investigation of public media resources, the following observations are reported. **Results**: Twenty-six victims died in the supermarket fire, and 20 who were admitted to local hospitals later succumbed. At Molo, 91 lives were claimed at the scene; 178 patients were admitted to various hospitals, 40 of whom died. **Conclusion**: The fires brought to light factors contributing to these events and their outcomes. In addition, it produced improvised solutions for resuscitation of mass casualties and the performance of emergency surgery with inadequate equipment and facilities.

Catastrophic fires have occurred in virtually every major city in the world. In the last century, there have been 73 such disasters in the United States alone. Experiences with these events have led to institution of fire regulations, creation of medical disaster response plans, and reshaping of governmental legislation.^1^–^3^ Nevertheless, burn disasters continue to pose logistical challenges in triage, management, and allocation of resources. When such fires occur in locations with limited resources, the challenges faced are significantly amplified. This was demonstrated in 2 tragic fires that occurred recently in Kenya.

Kenya is located on the eastern coast of Africa overlooking the Indian Ocean, with a population of more than 5 million. Its capital city, Nairobi, has a population of some 3 million. Occupying the Central Business District of Nairobi is a dense conglomerate of businesses and shopping centers (Fig 1).

## NAKUMATT DOWNTOWN SUPERMARKET FIRE

The following is a compilation of eye-witness accounts of the Nakumatt Downtown Supermarket Fire (January 28, 2009; with permission of Nation Media Group).

During a normally busy afternoon in the Central Business District, traffic crawls through streets congested with pedestrians. Customers and employees in the crowded Nakumatt Supermarket go about their business. A power transformer blows and the lights cut out momentarily, causing some bewilderment. Moments later, the backup generator can be heard as it is activated, rekindling the lights. As the people regain their bearings, the lights go out again, followed by a plume of thick smoke that is shrouded by darkness, but chokes all that were within its reach. What was generalized confusion instantly turns into widespread panic. The fire alarm sounds amid the screams. A few employees struggle with a fire hose as the flames spread from beneath the staircase, where the generator is housed on the ground floor. Then there is a series of explosions as the fuel tanks stored next to the generator detonate.

## VIDEO 1

**Figure:**
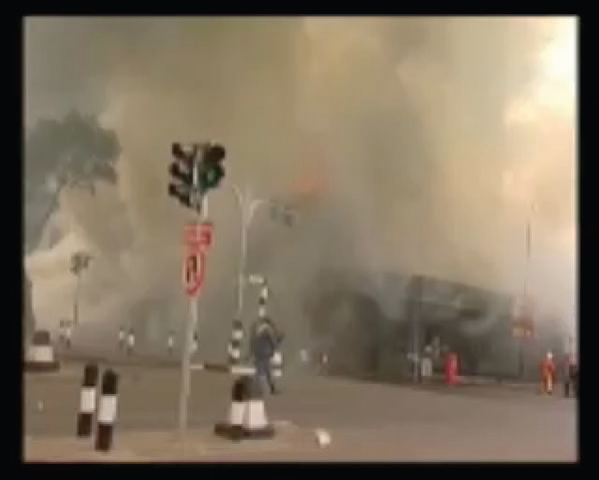
Video 1. Kenya Nakumatt Fire Movie

The Supermarket was located in Nairobi's Central Business District on Kenyatta Avenue. A historic building, it stood 3 floors high. The entire store burned to the ground together with the neighboring Alibhai Shariff Building. The fire started around 3:45 PM, on January 28, 2009, with workers and customers fleeing from the flames and smoke. As the fire spread to the neighboring buildings, gas tanks and containers filled with flammable chemicals in a hardware store ignited and fueled the already-uncontrollable blaze. Since the fire started on the ground floor beneath a stairway, dozens of people became trapped above and many sustained serious injuries when forced to jump from the upper levels.

**Figure 2:**
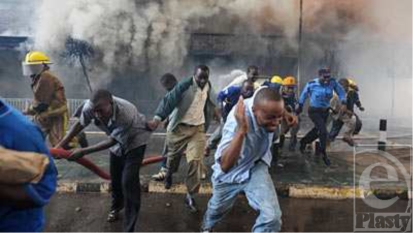
Customers and employees fleeing from Nakumatt Supermarket.

**Figure 3:**
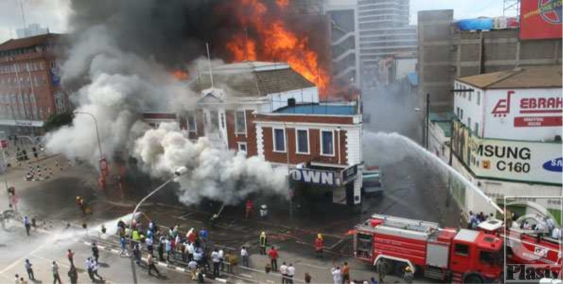
Fire brigade arriving, battling the Nakumatt Supermarket fire.

It took over an hour for firefighters to reach the blaze. The Nairobi City Council Fire Brigade, Kenya Police, Kenya Airports Authority Fire Fighters, G4S Fire Brigade, Kenya Airforce, Securex Fire, and Kenya Red Cross all eventually responded. Casualties were taken to Kenyatta National Hospital and surrounding Nairobi City Hospitals (Figs 2 and 3).

**Figure 4:**
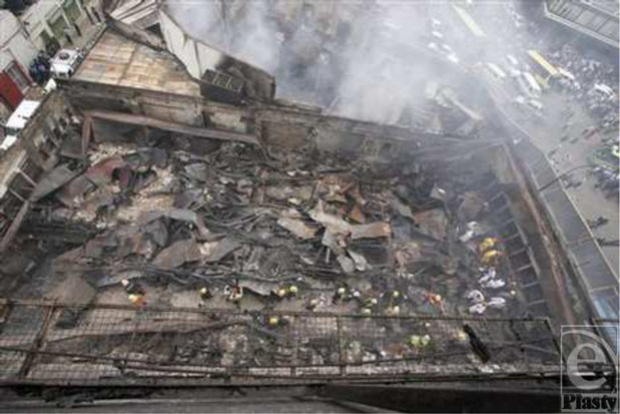
Search for victims of the Nakumatt Supermarket fire.

The fire was extinguished with difficulty, owing to the abundance of inflammable materials within the buildings and continued to burn for almost 3 days. Not until the arrival of the Kenyan Air Force firefighters with powdered carbon dioxide fire extinguishers would the fire ultimately be controlled. In the aftermath, 26 bodies were recovered from the ruins, most of them unrecognizable. Many critically ill patients would later succumb to their injuries, bringing the death toll to 46 (Fig 4).

## MOLO TANKER FIRE

A few days later, near the small town Sachang'wan, in the Molo District of the Rift Valley Province, a second tragedy unfolded. Around 7:30 PM, a tanker truck filled with approximately 42,000 L of petrol traveling from Mombasa overturned on the side of the Nakuru-Eldorat highway. The following is also a compilation of eye-witness accounts from survivors and observers of the events that occurred on February 2, 2009 (with permission of Nation Media Group).

As fuel spills onto the ground, word of the accident spreads and hundreds of nearby inhabitants rush to the scene. Drivers and motorcyclists call one another, and workers on nearby farms, villages, and shopping centers scurry to the site, as do dozens of children who are off school because of the weekend. With various containers and makeshift siphons in hand, there are frantic efforts to collect the spilt fuel. Some women with small children strapped to their backs are among those gathered. The crowd continued to enlarge as some proceed to drill holes into the tanker to expedite the collection process. Some even start charging a fee to collect the petrol. Soon thereafter, the local police and paramilitary General Service Unit arrive in attempts to control the unsettled crowd. As the people continued to scuffle for the spilling petrol, a cigarette was believed to have triggered the fire and subsequent explosion.

## VIDEO 2

**Figure:**
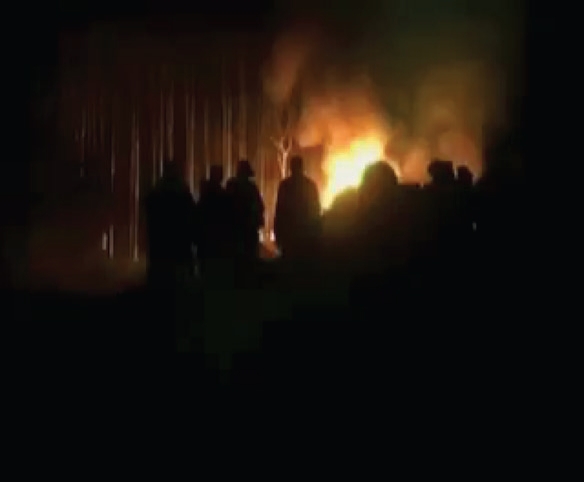
Video 2. Kenya Molo Fire Movie

Hundreds of people hovered around the tanker and standing in surrounding petrol-filled trenches were incinerated immediately. Hundreds more standing within 50 m of the truck also fell victim to the blast. The explosion set the surrounding forest ablaze, as well as several cars and motorcycles. Some of the burned victims tried to make their way through the wooded area to the nearby Molo River. Eight bodies were found at a nearby cattle trough as they attempted to douse the flames that consumed them.

**Figure 5:**
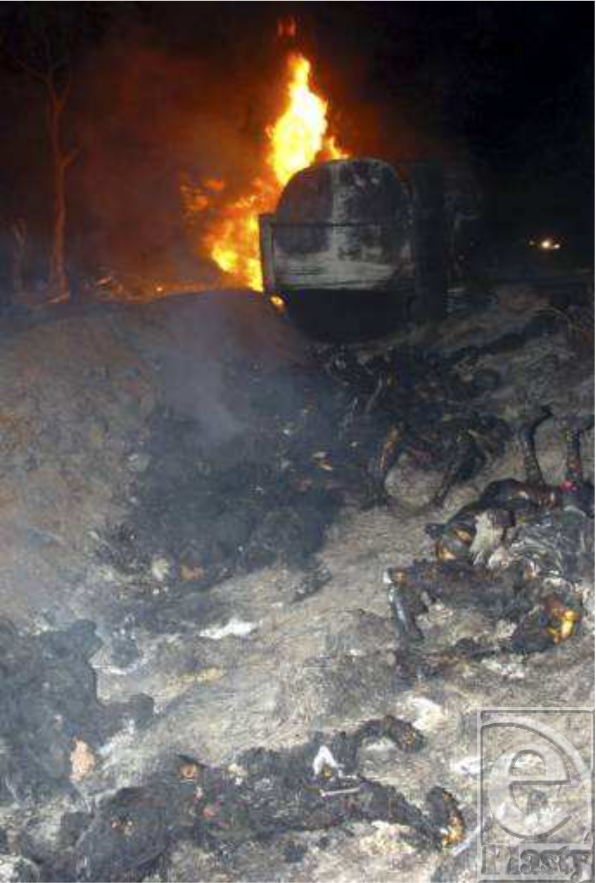
Victims of the Molo tanker fire.

**Figure 6:**
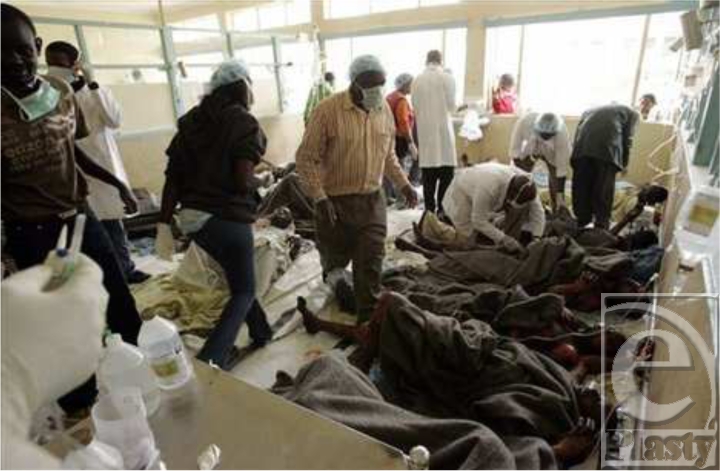
Resuscitation at the scene of the Molo tanker fire.

An estimated 91 victims were killed at the scene; many of them were women and children. Another 178 people sustained severe thermal injuries. Molo did not have its own fire engine. The Nakuru Municipal Council's firefighters did not arrive until an hour after the initial explosion. Rescuers who arrived at the scene were kept at bay by the intense heat, neither able to fight the fire nor able to help the injured victims. Most of the bodies were burnt beyond recognition (Figs 5 and 6).

A few ambulances and several onlookers loaded burnt survivors into their vehicles and transported them to the nearby Rift Valley Provincial General Hospital and Molo District Hospital. Ambulances were also dispatched from Nairobi, Kenyatta, and Aga Khan Hospitals from Nairobi, which were several hours away. The survivors were distributed to neighboring hospitals (Table 1). Unfortunately, these hospitals were still in the mid of dealing with the Nakumatt Supermarket fire tragedy. Eventually the death toll rose to 131 as more patients eventually succumbed to their injuries.

The Kenyan Army, Airforce, and Red Cross coordinated by the Ministry of Public Health and Medical Services collaborated to collect and deliver medical supplies and donated blood. They also facilitated the transfer of patients between the hospitals by helicopter. Teams of military healthcare workers were dispatched to the local hospitals to assist in treatment as well. The Kenyan government and Ministry of Public Health also put out a worldwide plea for aid, which was answered by The James Jordan Foundation. An additional group was also deployed from India.

## EXPERIENCE OF THE SURGICAL TEAM AT KENYATTA NATIONAL HOSPITAL

**Figure 7:**
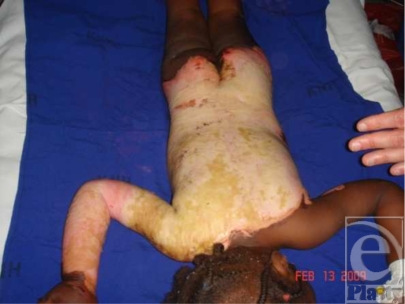
An 8 year-old female with infected burns to her trunk and right upper extremity.

Kenyatta National Hospital in Nairobi is considered the largest and most advanced hospital in Kenya. Their burn unit consisted of approximately 8 rooms and a dedicated operating room. Our team arrived 10 days after the fires and the wards were full, mostly of children and adolescents. It was a credit to the hospital that so many had survived; however, most of the patients with larger burns had extensive infected eschars (Fig 7).

**Figure 8:**
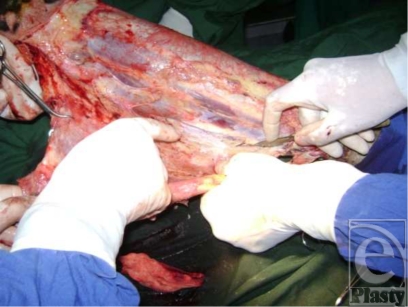
Fascial excision of leg performed with scalpel. A rubber glove is tied around the thigh as a tourniquet.

Emergency fascial excisions were performed as a matter of urgency, to remove as much necrotic tissue as possible and limit the sepsis. By modern standards, the conditions were meager. The medical supplies were extremely limited. Items that would routinely be thrown away in modern hospitals were recycled indefinitely. The sterile technique observed in contemporary operating rooms was difficult to perform. Essential equipment such as tourniquets, electrocautery, and powered instruments such as dermatomes were absent. Improvisations included the use of rubber gloves as tourniquets; excisions were carried out by sharp dissection and skin graft harvested with a Humby knife (Fig 8).

## VIDEO 3

**Figure:**
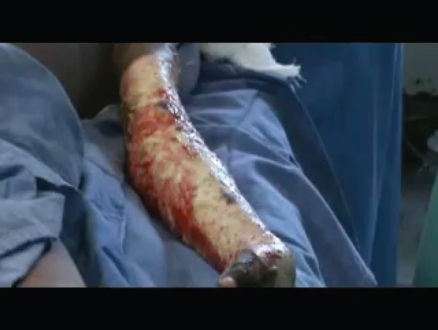
Video 3. Kenya Arm Excision Movie

## VIDEO 4

**Figure:**
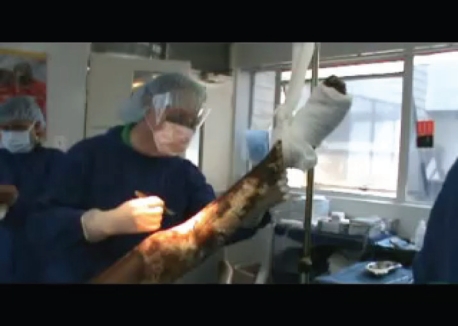
Video 4. Kenya Leg Excision Movie

## DISCUSSION

In the days following the tragic events, the public and media would express concern regarding the lack of fire preparedness and disaster response displayed by the city and government for both incidents. Molo, a town with a population of approximately 90,000, completely lacked a fire brigade, the nearest being an hour away in the neighboring town of Nakuru. The situation regarding the Nakumatt Supermarket was also scrutinized. In the capital city of Nairobi, with a population of more than 3,000,000, there was only 1 fully equipped fire brigade. Although the fire station was within the downtown area, it took over an hour for the arrival of firefighters due to poorly controlled traffic and a lack of a response plan. Survivors and witnesses of the supermarket fire suspected that the supermarket employees and security guards may have locked the doors to prevent looting, thus trapping customers in the building and preventing their escape. This was corroborated by the difficulties the firefighters encountered when trying to enter the building through these locked doors. Furthermore, the bodies that were recovered were hovered around the doors that normally served as exits. The hospitals were certainly overwhelmed by the sheer volume of casualties as well as the inability to access proper resources.

Deadly tanker fires are common in Africa. Recently, there have been similar incidents throughout Africa, causing hundreds of deaths. In March 2007, 98 people died from a tanker explosion in Nigeria.^4^ Similarly, in August 2008, more than 30 people died in a tanker explosion in Cameroon.^5^ In November 2008, more than 20 people died in yet another incident in Ghana.^6^ Barely 2 days after the Molo tanker fire, a derailed cargo train transporting detergent chemicals was emptied by nearby Kenyan residents of Nivasha. In another incident, merely 32 km from the site of the tanker fire, truck carrying maize collided with another vehicle. Again, people rushed to the scene to empty the fuel tanks of the vehicles.

There are several factors that may have contributed to the dire circumstances that surrounded these 2 incidents and their outcomes. As has been repeatedly proven, these looting practices create fatal situations. Many blame the overall state of poverty in Kenya as the driving force toward these desperate and dangerous acts of stealing fuel. However, it is believed to be much more complicated than that. Siphoning fuel from tankers along Kenyan highways has become relatively commonplace.^7^ Transporters are estimated to lose millions of shillings in this racket yearly. These activities are especially common along steep sections of Kenyan highways where large trucks carrying goods are forced to travel at slow speeds and stop frequently. The hazardous roads make the trucks prone to flipping, by accident or even deliberately. There is believed to be a whole network of cartels that collaborate with truck and train crews, to orchestrate these thefts.

There was certainly a deficit of firefighting personnel and equipment. The Molo District completely lacked a fire brigade. In the highly populous urban Nairobi, there is a single fire brigade that serves 3,000,000 people. Even when within several hundred meters of the Nakumatt Supermarket, the response time was greater than an hour. Furthermore, the manner in which the fire was fought was inappropriate due in part to lack of knowledge and firefighting resources. Although the claims of the exits being locked had not been confirmed, certainly keeping exits free of obstruction would facilitate escape.

It is extremely difficult to criticize the hospitals that are involved in these mass casualty situations. Even in the most industrially advanced countries with cutting-edge technologies and well-equipped medical centers, tragedies such as these would certainly exceed their resources as well.

An interesting observation, evident on documentation, was the manner in which many of these patients were resuscitated. Casualties were given plain drinking water for acute resuscitation. Paucity of equipment and staff prevented administration of fluids intravenously, as well as appropriate medication, or monitoring. Assuming that the patients are triaged appropriately and limited resources allocated accordingly, the easiest, fastest, and most cost-effective way of resuscitating burned patients may well be via oral resuscitation. This can also be used to supplement intravenous measures.^8^

Medical records of patients admitted to Kenyatta National Hospital were reviewed. All of them received a significant amount of water during the acute/resuscitative phase as well as the hypermetabolic phase. It was noted that all of the patients became hyponatremic, with serum sodium levels ranging from 123 to 130 mEq/L. The complications from hyponatremia, such as water toxicity and cerebral edema, were not evident.

Through tragic events such as these 2 catastrophic fires, many issues come to the forefront; questions are raised, regulations are evaluated, and criticisms are voiced. The perceived lack of disaster readiness in the medical and nonmedical emergency services has certainly come under scrutiny in the Kenyan public and media. Since these incidents, efforts led by the Ministry of Public Health and Medical Services are being made to enhance disaster preparedness and response. Sadly, many economic and social issues may continue to create these hazardous situations, ultimately undermining the promotion of disaster preparedness and response.

## Figures and Tables

**Figure 1:**
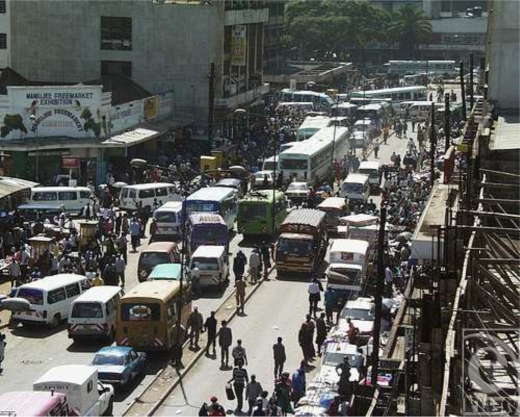
Crowded street traffic in downtown Nairobi.

**Table 1:** Distribution of casualties from the Molo Tanker Fire

Hospital	No. of victims
Molo District Hospital	112
Nakuru Provincial General Hospital	67
Kenyatta National Hospital	46
Kenyatta Hospital	28
Mater M. Hospital	12
Nairobi	3
Aga Khan	3
